# Pathway for Water Transport through Breathable Nanocomposite Membranes of PEBAX with Ionic Liquid [C_12_C_1_im]Cl

**DOI:** 10.3390/membranes13090749

**Published:** 2023-08-22

**Authors:** Ziqi Cheng, Shen Li, Elena Tocci, Giacomo Saielli, Annarosa Gugliuzza, Yanting Wang

**Affiliations:** 1 CAS Key Laboratory of Theoretical Physics, Institute of Theoretical Physics, Chinese Academy of Sciences, Beijing 100190, China; 2 School of Physical Sciences, University of Chinese Academy of Sciences, Beijing 100049, China; 3 National Research Council―Institute on Membrane Technology (CNR-ITM), Via Pietro Bucci 17C, 87036 Rende, Italy; e.tocci@itm.cnr.it; 4 National Research Council―Institute on Membrane Technology (CNR-ITM), Unit of Padova, Via Marzolo, 1, 35131 Padova, Italy; giacomo.saielli@unipd.it; 5 Department of Chemical Sciences, University of Padova, Via Marzolo, 1, 35131 Padova, Italy; 6 Center for Theoretical Interdisciplinary Sciences, Wenzhou Institute, University of Chinese Academy of Sciences, Wenzhou 325001, China

**Keywords:** ionic liquid crystal, PEBAX membrane, breathability, molecular dynamics simulation

## Abstract

Water transport through membranes is an attractive topic among the research dedicated to dehydration processes, microenvironment regulation, or more simply, recovery of freshwater. Herein, an atomistic computer simulation is proposed to provide new insights about a water vapor transport mechanism through PEBAX membranes filled with ionic liquid (IL) [C_12_C_1_im]Cl. Starting from experimental evidence that indicates an effective increase in water permeation as the IL is added to the polymer matrix (e.g., up to 85·10^−3^ (g·m)/(m^2^·day) at 318.15 K for PEBAX@2533 membranes loaded with 70% of IL), molecular dynamics simulations are proposed to explore the key role of IL in water transport inside membranes. The polar region composed of anions and cationic head groups of the IL is demonstrated to serve as the pathway for water transport through the membrane. Water molecules always stay near the pathway, which becomes wider and thus has a larger water-accessible area with increasing IL concentration. Hence, the diffusion coefficients of water molecules and ions increase as the IL concentration increases. The simulation provides useful indications about a microscopic mechanism that regulates the transport of water vapor through a kind of PEBAX/IL membrane, resulting in full agreement with the experimental evidence.

## 1. Introduction

PEBAX@2533 [80PTMO/PA12] is an elastomeric block copolyamide with high processability [1,2], mechanical strength [3], and high-performing permeability to vapors and quadrupolar and condensable gases [4,5,6,7]. These features make it particularly attractive and competitive for applications in wearable textiles [8], industrial equipment [4,9,10], microelectronics [11], and environmental protection [12]. This copolymer is widely used in the preparation of breathable films for regulation of microclimate [13,14], which is regarded as a micro-space air stream between two different neighboring gaps. The regulation of microclimate is of great importance in the preservation of works of art [15] since rapid changes in relative humidity can destroy or irreversibly compromise the integrity of cultural heritage. Similarly, a lack of balance between temperature and humidity can affect microclimate conditions in textiles [8,16,17], causing discomfort and chances of skin rashes, itching, and allergies. Last but not least, dehydration is also applied to preventing corrosion from condensed water in source gas streams [18,19]. The membrane technology can provide suitable and intelligent solutions and benefits in the fields wherein removal or regulation of water vapor concentration is desired [20]. Despite the fact that a large number of materials have been proposed to intensify the transport of water vapor through the PEBAX membranes [14,21,22,23,24], most of them do not satisfy today’s large demand of adapting themselves according to external changes in a fast and reversible way. This aspect is relevant to the design of intelligent systems that could restore and maintain constant desired microenvironments in a reversible and highly reproducible way.

Ionic liquids (ILs) are room-temperature molten salts with unique properties such as negligible vapor pressure, good thermal stability and non-flammability, as well as high ionic conductivity and a wide window of electrochemical stability [25,26,27]. They also have the ability to dissolve most organic and some inorganic materials as well as biopolymers [28]. Ionic liquid crystals (ILCs), namely ILs in their liquid crystal state, are a preferred family of materials for their reversible assembling ability [29,30]. Long-chain imidazolium-based ILs are well-known compounds capable of forming ILCs and continuously receive a great deal of attention as materials that combine the unique solvent properties of ILs with the long-range partial order of LCs [31]. Effective use of ILCs as electrolytes in dye-sensitized solar cells [32,33], electro-fluorescence switches [34], electrolytes for Li-ion batteries [35], and electrochemical sensors [36], to mention but a few, has been clearly demonstrated. When they are embedded in polymeric membranes, ILCs can play a key role since in the ionic fluid phase they have special conductive properties of mass and charge, while in the ordered fluid phase they have preferential directions that enable fine tuning of the transport properties of the geometry and structure of the polymer and of external parameters [37].

A lot of studies have been dedicated to the analysis of structure and dynamics in bulk ionic liquids [38,39,40,41,42,43], whilst the number of papers dedicated to the confinement of these materials in organic and inorganic matrixes and to changes in IL structure and interfaces is somewhat limited [44]. When filling polymeric matrixes with ILCs, changes in the final properties of the nanocomposite systems can be experimentally detected by suitable tools [45]; however, the relationship between the low dimensionality of interface systems and fluidity of the interfaces becomes quite difficult to understand. Indeed, the shape of the ILC interfaces and the orientation and structure of fluid molecules constantly change, producing nanoscale effects whose amplification is perceived on a larger scale. As known, IL compounds have a unique chemical structure with a large amount of possible organic cation and anion species from which to select. These ions can interact with organic polymers and inorganic nanofillers, and their intermolecular interactions can modify the polymer nanocomposites and enhance the interfacial connection between the polymer matrix and the nanofillers [46]. The modification of nanofillers by IL is a promising method to prepare multifunctional polymer nanocomposites, which can be regarded as a practical route for the development of adaptable and monitorable systems [44]. Much experimental evidence has shown that the addition of IL to PEBAX can lead to the formation of more amorphous and less crystalline membranes [47,48] due to the fact that ILs increase the tendency of polymers to form new bonds and form a homogeneous mixture with the IL. Ample experimental work has shown that membranes of PEBAX or other polymers with ILs have excellent gas permeability and selectivity [49,50,51,52]. In various types of ILs, imidazolium-based ILs are often used to modify nanofillers because they work in a wide variety of chemical structures [53].

In this work, we perform atomistic molecular dynamics (MD) simulations that provide new insights into the reorganization of 1-dodecyl-3-methylimidazolium chloride ([C_12_C_1_im]Cl, abbreviated as C_12_) confined in PEBAX@2533 elastomeric membranes and into the establishment of local interfacial forces and tunneling events that assist water mass transfer through nanocomposite matrixes. As a case study, we analyze PEBAX@2533 membranes embedding a large amount of [C_12_C_1_im]Cl, which exhibit an increasing capability to transfer a large quantity of water vapor with concentration and temperature at high reproducibility and in a reversible way. The choice of PEBAX as a host matrix is due to the fact that large amounts of nanofillers can be embedded in a nanofilm without compromising manageability thanks to its elastomeric properties.

Starting from experimental evidence of the changes in water permeability, our intent is to study, at the molecular scale, the relationships established between IL concentration and diffusing water molecules, in order to assess the behavior of ILs when confined in polymeric networks and approaching polar penetrant such as water. This study provides new useful insights about the action of ionic groups in water transport while also suggesting the ability of IL to self-assemble into somewhat ordered regions when constrained in an elastomeric host polymer network. Through our simulation results, we have found that the polar parts of the ILs form a polar network in the membrane, serving as a pathway for water to penetrate. The pathway becomes wider as the concentration of IL in the membrane increases; thus, a larger water-accessible area is provided and the diffusion of water molecules becomes faster. This effect increases further with temperature due to thermal motion of water molecules, which facilitates the diffusion through polar pathways. Undoubtedly, the choice of confining an IL in an elastomeric polymer network such as PEBAX provides more freedom of movement and rearrangement, yielding high reproducibility and reversibility, especially at higher temperatures. Moreover, with molecular mechanisms clarified, thermosensitive materials such as ILs are expected to provide a great chance of synchronizing transport properties with the external environment.

This study is therefore a critical and due step to identify driving forces for the design of responsive breathable membranes necessary for microclimate regulation and controlled dehydration. More specifically, this study can be regarded as a preliminary step towards controlling, at the molecular scale, water permeation through membranes for the development of intelligent devices that enable the regulation of humidity in microenvironments.

## 2. Experimental and Simulation Methods

### 2.1. Materials

The thermoplastic elastomer poly(ether-block-amide) (PEBAX@2533, Arkema, Milan, Italy) was used as a precursor of dense membranes (10 wt.%), and 1-dodecyl-3-methylimidazolium cation of [C_12_C_1_im]Cl was purchased from IoLiTec (Heilbronn, Germany) and used as the nanofiller at different concentrations of 30, 50, and 70% (*w*/*w*), and 1-Proponal (PrOH) and *n*-butanol (BuOH) (99.5%, Carlo Erba, Milan, Italy) were used in a mixture (1:3 *v*/*v* %) to dissolve polymer and nanofiller. All materials were used as received.

### 2.2. Membrane Preparation and Breathability Tests

Nanocomposite membranes were prepared through the blend of an elastomeric poly(ether-block-amide) (PEBAX@2533) with -dodecyl-3-methylimidazolium cation of [C_12_C_1_im]Cl according to dry phase inversion [22]. The membranes were prepared by dissolving the IL at different contents in a mixture of PrOH/BuOH, and the polymer was then added to homogeneous solutions under vigorous stirring. Clear solutions were poured in Petri glasses, and the solvents were evaporated under controlled environmental conditions (*T* = 293.15 K and RH < 50%) to obtain membranes with a thickness of 50 μm. The membranes were air-dried for 4–5 days at room temperature and then stored in an oven at 40 °C for 3 days and under vacuum at room temperature for a further 7 days to remove solvent traces. The membrane breathability, herein expressed as water permeation (g·m)/(m^2^·day), was tested within a range of temperatures (298.15–318.15 K) according to the right cup method (ASTM E96B) [8,54]. Water breathability was expressed as the amount of water transmitted over time through a specific area of the membrane. The breathability was normalized per the thickness of each membrane for comparison.

### 2.3. Simulation Methods

**Figure 1:**
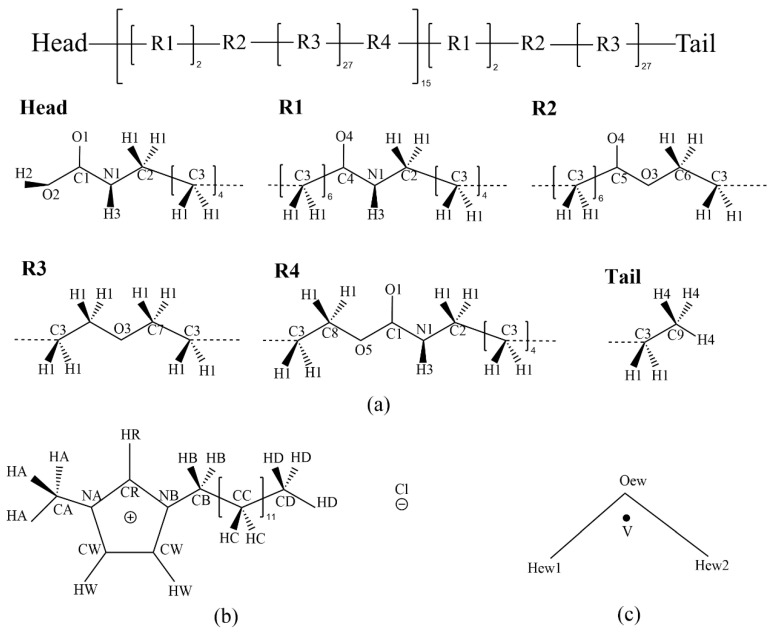
Molecular structures of PEBAX (**a**), [C_12_C_1_im]Cl (**b**), and TIP4P/EW water model (**c**).

All-atom MD simulations were performed to study the structural and dynamical characteristics of water molecules in PEBAX membranes filled with [C_12_C_1_im]Cl. The molecular structures are shown in Figure 1.

The force field parameters for PEBAX and [C_12_C_1_im]Cl were all taken from the OPLS-AA force field [55,56]. The partial charges of all the atoms in PEBAX and [C_12_C_1_im]Cl were calculated with the RESP method [57]. As suggested by Chaban et al. [58], the net charges of [C_12_C_1_im]Cl were reduced by a factor of 0.8 to conveniently mimic ion polarizability. All partial charges we have calculated are listed in Table 1, and the associated atom names are marked in Figure 1. The TIP4P/EW force field was adopted to model water molecules (Figure 1c), in which the position of the virtual site ‘V’ $r_{V}=r_{\mathrm{Oew}}+a\left(r_{\mathrm{Hew}1}-r_{\mathrm{Oew}}\right)+b\left(r_{\mathrm{Hew}2}-r_{\mathrm{Oew}}\right)$ with a=b=0.106676721, where $r_{\mathrm{Oew}}$, $r_{\mathrm{Hew}1}$, and $r_{\mathrm{Hew}2}$ are the positions of the oxygen and hydrogen atoms. All force field parameters of TIP4P/EW are listed in Table 2, from which the effective diameter of a water molecule can be estimated as about 2.8 angstroms [59]. To simulate membranes with different concentrations of [C_12_C_1_im]Cl, three different systems were constructed, whose data are summarized in Table 3. The number of water molecules remains 1/3 of [C_12_C_1_im]Cl ion-pair numbers, and five additional systems with a molecular ratio of water to C_12_ of 1/3, 2/3, 1, 2, and 10 were also simulated, keeping the 30% *w*/*w* as a typical C_12_ concentration (Table 3). Six BEBAX chains are enough for investigating their local structural features with respect to water and IL molecules.

All the systems simulated in this work are in a cubic box with periodic boundary conditions applied to all three dimensions. A cutoff distance of 12 Å was used to treat the van der Waals (VDW) and the real part of the electrostatic interactions, and the particle-mesh Ewald method [60] was applied to calculating the electrostatic interactions. For three different membranes without water, initial configurations were prepared by a simulated annealing procedure in an *NVT* ensemble for 6 ns from 1000 K down to 600 K, continuously. Another simulated annealing procedure in an *NPT* ensemble was then followed with the following sequential steps: 4 ns at 600 K and 1 bar, 6 ns at 500 K and 100 bar, 6 ns at 450 K and 50 bar, 6 ns at 400 K and 1 bar, 10 ns at 350 K and 1 bar, and 10 ns at 298 K and 1 bar.

To prevent water molecules from erroneously affecting the structure of the nanocomposite films at high temperatures, water molecules are randomly inserted into the membrane configuration at 300 K and 0.5 ns in an *NPT* ensemble to obtain the appropriate system size. Water molecules are then fully mixed with the membrane in an *NVT* ensemble after lowering the temperature from 500 K down to 450, 400, 350, 328, and 298 K, with a simulation duration of 1 ns at each temperature. In order to obtain accurate kinetic data, a 60 ns simulation in an *NPT* ensemble was performed at 298 K and 318 K, respectively, and data were sampled in the last 20 ns simulation.

In these runs, the system temperature and pressure were kept constant by using the Nosé–Hoover thermostat [61] with a time constant of 0.5 ps and the Parrinello–Rahman barostat [62] with a time constant of 2 ps, respectively. All the simulations were performed with GROMACS 2020 software [63] with a time step of 1 fs.

## 3. Experimental Results

**Figure 2:**
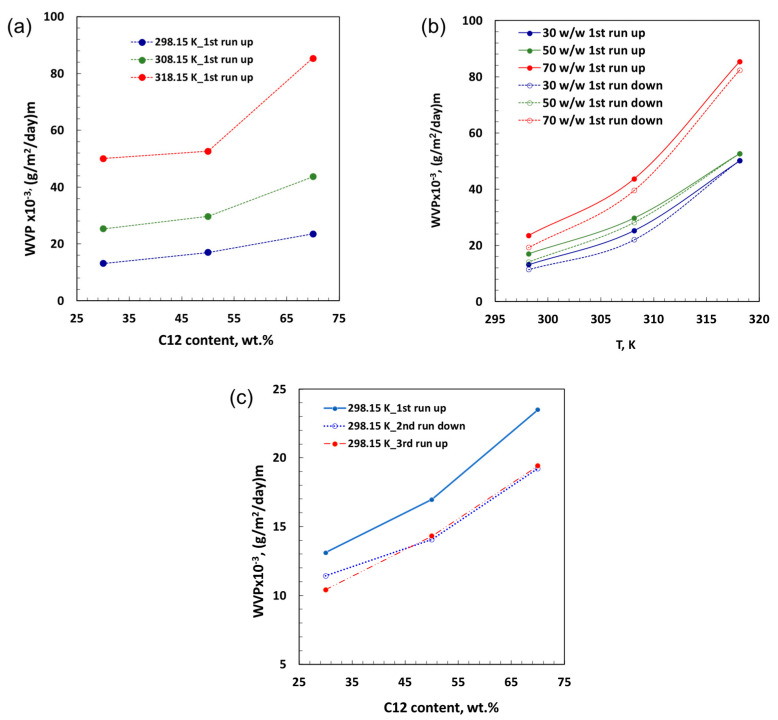
(**a**) Water permeation through PEBAX membranes containing different amounts of [C_12_C_1_im]Cl. (**b**) Estimation of water permeation with running uphill and downhill temperatures. (**c**) Reproducibility of water permeation properties estimated at 298 K for all nanocomposite membranes.

Dense nanocomposite PEBAX membranes filled with [C_12_C_1_im]Cl were prepared via dry phase inversion with the intent to investigate how ILCs can affect water vapor transport through them. Changes in permeation, namely breathability, were investigated according to the right cup method procedure [8,54], and membranes filled with three different amounts of filler (30, 50, 70 *w*/*w*) were tested by running the temperature from 298.15 to 318.15 K and then down to 298.15 K again (Figure 2). Interestingly, the increase in the [C_12_C_1_im]Cl content produces amplified water transport, which is further raised with temperature. This effect appears to be more marked at the highest concentration of filler, indicating a wider assistance to water vapor permeation (Figure 2a). Moreover, the reversibility of the process can be detected as the temperature decreases from 318.15 to 298.15 K (Figure 2b), suggesting the IL as a powerful choice for making the membranes responsive to changes in temperature.

In all cases, a small amount of hysteresis can be observed between running uphill and downhill temperatures due to a probable reorganization of the materials assembled inside an elastomeric membrane. As proof, the third run uphill at 298.15 K indicates a substantial overlapping of permeation properties for the membranes containing different amounts of [C_12_C_1_im]Cl (Figure 2c). This implies a certain fluidity of ILC in this kind of constrained polymeric system and a great freedom to rearrange itself quickly and reversibly. On the other hand, the elasticity of the host polymer is expected to facilitate the self-assembly of the IL during confinement as well as its rearrangement with temperature (Figure 2b).

It is well known that PEBAX@2533 has a great ability to embed large amounts of nanofiller [13,64] and dissolve polar penetrants through solution–diffusion mechanisms [65,66]. Considering the elastomeric features of this polymer [67], this behavior is not surprising and suggests a major accessibility of water molecules to hydrophilic sites, wherein they can be allocated temporarily. An increase in thermal motion is further expected to allow water to be accessible to a larger number of sorption regions and diffuse itself through broader fluctuating free gaps generated by higher mobility of the polymer segment chains. In a previous work [46], the DFT calculation demonstrated that a low concentration of a non-ionic organic nanofiller in PEBAX causes a competition of interaction energies determining higher availability of polymer polar moieties as a domino effect, while at a higher concentration the polar moieties are saturated and disallow water sorption [46].

In the present study, a significant increase in water permeation is instead observed with raising IL concentration. However, it is crucial to understand how and if the IL self-assembles in a constrained environment and which chemical moieties are involved in water transfer. To understand which forces and events address the behavior of water molecules during penetration in mixed matrices, a computational investigation at the micro- and nano-scale is hence necessary.

With this purpose, MD simulations were performed to corroborate this experimental evidence, serving as a case study and shedding light on the role that polar intermolecular interactions and the charged pathway play in the transport of water molecules through polymeric matrixes containing a typical IL such as [C_12_C_1_im]Cl.

## 4. Simulation Results

### 4.1. Water Pathway Formed by IL in Nanocomposite Membrane

**Figure 3:**
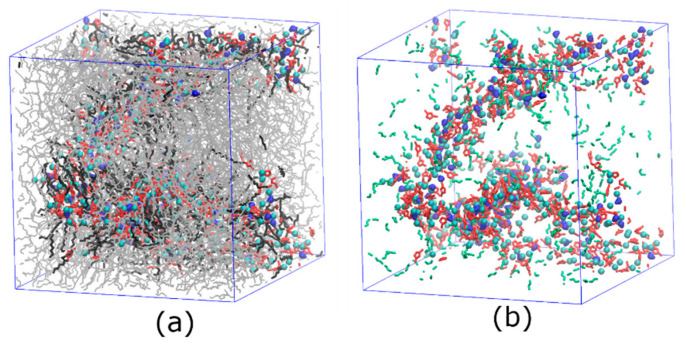
The snapshots of membranes with PEBAX and 30% *w*/*w* [C_12_C_1_im]Cl. The mole ratio of water to [C_12_C_1_im]Cl is 1:3. (**a**) All components. (**b**) Polar groups (head of cation, Cl^−^, water, and amide) only. The gray lines are PEBAX chains, the black lines are [C_12_C_1_im] side chains, the red rings are cationic head groups, the cyan balls are chloride ions, the blue beans are water molecules, and the green sticks are amide bonds in PEBAX.

**Figure 4:**
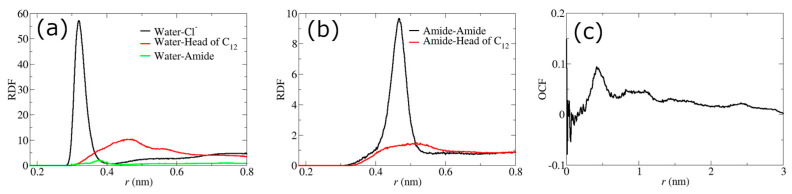
RDFs of (**a**) water with Cl^−^, head of [C_12_C_1_im], and amides; (**b**) amides with amides and head of [C_12_C_1_im]; (**c**) orientational correlation function of [C_12_C_1_im] side chains, respectively, for the membrane with PEBAX and 30% *w*/*w* [C_12_C_1_im]Cl at 298 K.

After the initial build-up structures of PEBAX filled with [C_12_C_1_im]Cl and water were equilibrated, the rearrangements of these molecules inside simulation boxes were examined. It was found that the IL cations were reorganized in designated regions compared to the structures of pure [C_12_C_1_im]Cl, orienting the polar head towards the core of the agglomerates composed of anions and cationic head groups, whereas the hydrophobic tails are oriented outwards (Figure 3a,b) due to the large amount of hydrocarbon chains in both [C_12_C_1_im]Cl and PEBAX that tend to mix with each other. Water molecules are more concentrated around Cl^−^ and the cationic head groups that form the water pathway, and only a few are present in the polymer region (Figure 3b and Figure 4a). On the other hand, amides tend to aggregate with each other, which may promote the cross-linking of polymer chains and thus improve the strength of the membrane (Figure 3b and Figure 4b). Moreover, the correlation between amides and the head of [C_12_C_1_im] (Figure 4b) improves the interfacial interaction and filler–polymer interface compatibility in the blend membrane. As ILs can form hydrogen bonds with the PA hard segments of PEBAX [68], the enhanced hydrogen bonding leads to better dispersion of the filler in the polymer.

It is worth mentioning that although the pure [C_12_C_1_im]Cl system exhibits an ILC state [69] in the temperature range of this study, due to the similarity between the alkyl cationic side chains of [C_12_C_1_im]Cl and PEBAX, the side chains themselves do not have long-range orientational correlations (Figure 4c), indicating that the ILs do not form the ILC structure inside PEBAX.

From the above simulation results, we conclude that the polar parts of the IL, composed of anions and cationic head groups, form a continuous polar water pathway inside PEBAX, which facilitates the transport of water molecules through the nanocomposite membrane of PEBAX with [C_12_C_1_im]Cl. The PEBAX polymers do not participate directly in forming the water pathway.

### 4.2. Influence of [C_12_C_1_im]Cl Concentration and Temperature

**Figure 5:**
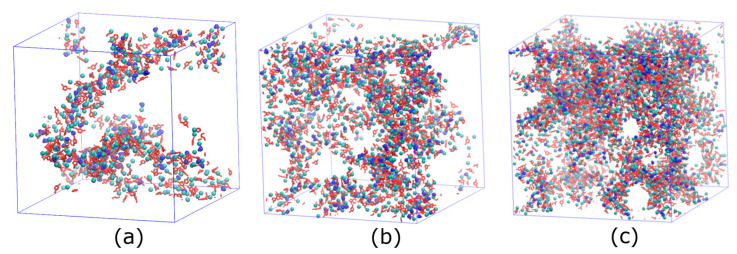
The snapshots of PEBAX membranes with 30% *w*/*w* (**a**), 50% *w*/*w* (**b**), and 70% *w*/*w* (**c**) [C_12_C_1_im]Cl at 298 K. Red rings represent cationic head groups, cyan balls represent chloride ions, and blue beans represent water molecules.

**Figure 6:**
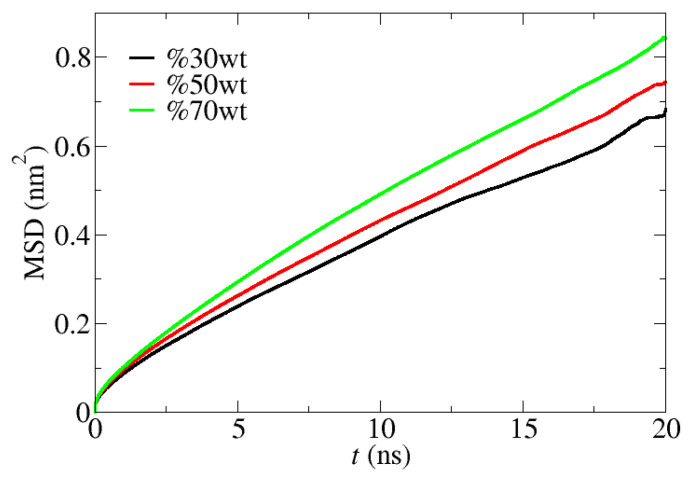
Mean square displacements for water molecules in the PEBAX membranes with 30% *w*/*w*, 50% *w*/*w*, and 70% *w*/*w* [C_12_C_1_im]Cl, respectively, at 298 K.

The network formed by the polar region of [C_12_C_1_im]Cl becomes larger with increasing [C_12_C_1_im]Cl concentration (Figure 5). Figure 6 shows the mean square displacements (MSDs), defined as $MSD\left(t\right)=\langle {\left|r\left(t_{0}+t\right)-r\left(t_{0}\right)\right|}^{2}\rangle$, where t is the time interval, $r\left(t_{0}\right)$ is the position of the atom at time $t_{0}$, and 〈⋯〉 denotes the ensemble average, for water molecules with different IL concentrations at 298 K. It can be seen that water molecules diffuse faster in the membrane with a larger IL concentration due to the fact that the polar region of the IL is larger. The smoothness of the MSD curves indicates that the simulation time is long enough to obtain reliable dynamics. The diffusion coefficient for certain types of atoms can be calculated by fitting the slope of the corresponding MSD as D=MSD/6t.

With the diffusion coefficients listed, Table 4 indicates that the diffusion of all molecules, i.e., water, Cl^−^ and N1, increases with the IL concentration, that the diffusion of water is much greater than for Cl^−^ and N1, and that the diffusion coefficients of chloride ion and water molecules increase with temperature significantly and are close to those in an aqueous solution at 318 K.

The above results can be understood as follows. Since water molecules always tend to stay in the polar region of [C_12_C_1_im]Cl, a higher concentration of [C_12_C_1_im]Cl in the nanocomposite membrane leads to a larger accessible area of polar water pathway and thus to faster diffusion of water through the hydrophilic sites. On the other hand, the polar water pathway assists the diffusion of water to provide an amplification of water transfer through the nanocomposite membranes. Additionally, because the thermal motion of water molecules increases with temperature, it is easier to move through the polar region at a higher temperature, which was also confirmed by our experimental evidence.

### 4.3. Influence of Water Concentration

**Figure 7:**
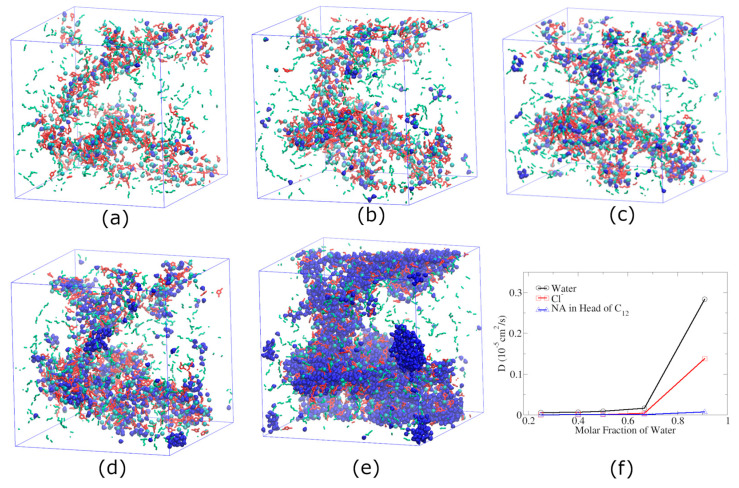
Snapshots of miscible membranes with PEBAX and 30% *w*/*w* [C_12_C_1_im]Cl at 298 K, whose mole ratios of water to [C_12_C_1_im]Cl are 1:3 (**a**), 2:3 (**b**), 1:1 (**c**), 2:1 (**d**), and 10:1 (**e**). The gray lines represent [C_12_C_1_im]Cl side chains, red rings represent cationic head groups, purple balls represent chloride ions, blue beans represent water molecules, and green beans represent amide bonds in PEBAX. The other parts of PEBAX are not shown. (**f**) Diffusion coefficients of water, Cl^-^, and the IL cationic head group at 298 K.

To understand how water concentration influences the polar water pathway described above, we simulated the nanocomposite membrane with PEBAX and 30% *w*/*w* [C_12_C_1_im]Cl along with a various number of water molecules at *T* = 298 K. As can be seen in Figure 7a–e, most water molecules were distributed in the polar region of [C_12_C_1_im]Cl. Moreover, as shown in Figure 7e, when water molecules are excessive, they will gather together to form droplet-like local structures rather than being distributed almost evenly and individually along the polar water pathway.

The diffusion coefficients of water molecules and chloride ions change slowly when the number of water molecules is small (Figure 7f). However, when the number of water molecules is large enough to form droplets, the diffusion coefficients of chloride ions and water molecules increase significantly and are close to those in an aqueous solution.

The tendency of the diffusivity of components in the system changing with the concentration of water molecules (Figure 7f) is basically consistent with the research results of Jiang et al.’s work [70] on water molecules in pure ILs, which reveals that the diffusion of water and ions is very slow but increases significantly when the water content is greater than 50% mole fraction.

These results indicate that when the water concentration is relatively low, water molecules distribute evenly and individually along the water pathway, the structural topology of the membrane is not altered, and the dynamics increase almost linearly with water concentration; when the water concentration is so high that water molecules are excessive, water molecules tend to form local water droplets, and correspondingly, the diffusivities of water molecules and anions increase drastically.

## 5. Conclusions

In [C_12_C_1_im]Cl and PEBAX nanocomposite membranes, at the nanometer scale, charged anions and cationic head groups of [C_12_C_1_im]Cl form a continuous polar network. Most water molecules disperse in the polar region of [C_12_C_1_im]Cl, and only very few are distributed near amide regions of the polymer. Therefore, we conclude that the polar region of the IL composed of anions and cationic head groups forms a water pathway for water to be transported through the IL-based polymeric membranes, and the PEBAX polymers do not participate in the water pathway directly. When the IL concentration increases, the polar network becomes larger, providing more hydrophilic sites where water molecules can be allocated. Consequently, the diffusion of water molecules increases with IL concentration and also increases with temperature due to faster thermal motion. The increase in water concentration changes the structural and dynamical properties of the system almost linearly when water molecules are not excessive, and drastically thereafter.

The above results suggest that the presence of hydrophilic [C_12_C_1_im]Cl regions inside polymer networks improves water adsorption and diffusion, yielding amplification of the mass transfer through the membrane, in full accordance with the experimental evidence. This study is a preliminary investigation on behavior of an IL in constrained host polymer matrices wherein water molecules are diffused. It provides useful indications about the behavior of materials confined in predefined volumetric space, suggesting that these kinds of materials are promising for realizing thermo-regulated permeable membranes.

## Figures and Tables

**Table 1:** The partial charges of PEBAX and [C_12_C_1_im]Cl.

Atom Name	Partial Charge/e	Atom Name	Partial Charge/e
C1	0.885	O1	−0.585
C2	0.104	O2	−0.612
C3	−0.106	O3	−0.396
C4	0.642	O4	−0.531
C5	0.702	O5	−0.266
C6	0.119	O6	−0.414
C7	0.027	N1	−0.699
C8	−0.159	NA	0.176
CA	−0.28	NB	0.176
CB	−0.136	H1	0.053
CC	−0.096	H2	0.423
CD	−0.192	H3	0.378
CR	−0.072	H4	0.064
CW	−0.192	HA	0.144
Cl	−0.8	HB	0.144
HW	0.216	HC	0.048
HR	0.168	HD	0.064

**Table 2:** Force field parameters of the TIP4P/EW water model.

Atom Name	Partial Charge/e	
Oew	0	
V	−1.04844	
Hew1	0.52422	
Hew2	0.52422	
**Valence bond**	**Bond length/nm**	**k_bond_/kJ mol^−1^ nm^−2^**
Oew-Hew1	0.09572	502,416.0
Oew-Hew2	0.09572	502,416.0
**Valence angle**	**Angle/** **°**	**k_angle_/kJ mol^−1^ rad^−2^**
Hew1-Oew-Hew2	104.52	628.02

**Table 3:** Number of different molecules for the simulated systems.

[C_12_C_1_im]Cl Concentrations in Weight	PEBAX Chains	[C_12_C_1_im]Cl Ion Pairs	Water Molecules	Water/C_12_ Ratio
30%	6	375	125	1/3
50%	6	870	290	1/3
70%	6	2040	680	1/3
30%	6	375	125	1/3
30%	6	375	250	2/3
30%	6	375	375	1
30%	6	375	750	2
30%	6	375	3750	10

**Table 4:** Diffusion coefficients of water molecules, Cl^−^, and the N1 atoms in [C_12_C_1_im].

Diffusion Coefficient 10^−5^ cm^2^/s		30 *w*/*w*%	50 *w*/*w*%	70 *w*/*w*%
Water molecules	298 K	0.00520	0.00563	0.00658
	318 K	0.01026	0.01449	0.01718
Cl^−^	298 K	0.00032	0.00048	0.00062
	318 K	0.00083	0.00139	0.00182
N1	298 K	0.00038	0.00051	0.00060
	318 K	0.00072	0.00118	0.00163

## Data Availability

Not applicable.
